# On the Natural History and Simple Treatment of Wounds

**Published:** 1846-07

**Authors:** Isaac Gilchrist


### II. ON THE NATURAL HISTORY AND SIMPLE TREATMENT OF WOUNDS.

By Isaac Gilchrist, m.d.

[Although we hope the practice described and recommended in the following paper is that which, in its main features, is now adopted very generally by enlightened surgeons in this country, its renewed promulgation in a more formal shape cannot fail to be useful. The natural treatment here illustrated is far from being universally followed; the old system of impertinent interference with Nature in all her ways being that still adhered to by many practitioners.*

In the present medical crisis, we, moreover, reckon it of especial importance that the attention of physicians should be directed to the curative activity of Nature in surgical cases, where the whole of the processes are subjected to the senses. If we find Nature capable of doing such great things, alone, or with that little help which scientific art deems it right to apply, on the surface of the body, we might reasonably infer that similar results would ensue under similar circumstances in the interior of the body; and such, assuredly, is the fact. But we see also, in the one case as in the other, that, although the actual worker of the cure, Nature stands often in need of the assistance of art to put her in the right road, to remove obstacles from her path, and, occasionally, to supply instruments which she does not herself possess. And in doing this the physician and surgeon have their high and true calling, and may

* For a graphical account of this "old system" see Mr. Liston's admirable 'Practical Surgery,' p. 31, fourth edition. And it is but justice to this distinguished surgeon to say, that much of the modern improvement in this department of surgical treatment is owing to his precepts and example.

always find ample and legitimate employment. All that Nature demands in such cases is, that Art should not, like Dr. Johnson's patron, "encumber her with help."]

---

*To the Editor of the British aud Foreign Medical Review.*

Woodside, Aberdeen, 26 May, 1846.

Sir,—Allow me to express to you the extreme satisfaction I have recently enjoyed from the careful perusal of two articles which appeared in the two numbers, for the present year, of the British and Foreign Medical Review, on the present state and anticipated reformation of the Medical Profession. I have been fortunate enough to have been, by certain appointments, very extensively engaged in the practice of our art during the last seven years, and my experience in the observation of disease has convinced me of the soundness of your views. In fact, I have for a considerable time been putting them to the test of experiment. The accompanying paper embodies the result of that experiment in a particular branch of the science. At the request of some of my brethren, who have witnessed the success of the simple treatment therein detailed, I had intended preparing a paper for some of the other journals; but, after reading your articles on the general subject, I thought that my observations on one division of it might with more propriety be sent to you. I am, Sir,

Yours, most respectfully,

Isaac Gilchrist.

---

In Sir John Herschel's Discourse on the Study of Natural Philosophy, it is stated that, " Art is the application of knowledge to a practical end. If the knowledge be merely accumulated experience, the art is *empirical;* but if it be experience reasoned upon, and brought under general principles, it assumes a higher character and becomes a *scientific art.*" It is humiliating indeed to consider that, in this age of boasted scientific advancement, the practice of medicine and surgery must be allowed to belong more to the former than the latter division of art. Has the progress of disease in the human body been studied in the same philosophical spirit as the processes observed in the external physical world? Have we kept in view that the laws of Nature comprehend in their dominion the course of a fever and the reparation of a wound, as well as the fall of an apple? Why have we long since ceased to struggle in the pursuit of the alchemists; and why do we exercise our ingenuity in paths of discovery different from that followed in the search after the perpetual motion? It is because the study of the laws of Nature has shown us how to avoid impossibilities.

Let us remember also that a knowledge of these same laws enables us " to accomplish our ends in the easiest, shortest, most economical, and most effectual manner. "It is only when the natural history of disease is studied philosophically that we can arrive at any real principles of therapeutics. The treatment of disease will only then be conducted upon sound principles when we know what can be " accomplished by Nature, and under what circumstances her operations may proceed with the greatest facility." When this is the case, there will be no more room for the craft and mystery of empiricism. Disease will not be considered an entity which must be driven out of the system by this medicine or by that.

The more unexplained any grievance is, the more numerous are the remedies usually proposed for its removal, and the greater the opportunity offered for the excitement of the strongest and blindest faith. Hence the reliance in former days upon charms, amulets, and incantations, and in our own, upon doctrines and systems equally baseless and absurd. Hence the expectation on the part of the patient that his medical adviser should *do a great deal* towards the removal of his disease, not knowing that certain salutary, natural processes may, by rash interference, be counteracted. Bodily disease doubtless produces impatience, and impatience too often produces imprudence; and hence Shakspeare's reproof:

> "How poor are they that have not patience!
> What wound did ever heal but by degrees?
> Thou know'st we work by wit, and not by witchcraft,
> And wit depends on dilatory time."

Thus the powers of Nature being overlooked, and all sorts of remedies being proposed as capable of combating with the unseen and unknown evils that oppress us, what else can be expected but that the boldest and most presumptuous treatment shall be accepted on the part of the impatient sufferer?

In another department of art man is content to prepare the ground and throw in the seed, relying implicitly on the laws of Nature that in due season the earth shall display its fruits; but he cannot, it would appear, exercise his faith in the same laws, when flesh and blood are revealing the processes of growth and reparation in the human body. Instead of watching, in the patience of hope, the operations of Nature, and learning with how very few medicines—or it may be with none—disease may be treated, we have been busy in the use of innumerable remedies (so called) and every variety of appliance, blindly believing that when a disease has been removed, the result was owing to the medication adopted. The various kingdoms of nature have been ransacked, every substance which she presents or which chemistry can form from her materials, has been tried in the practice of medicine. Each of these articles, moreover, must be tortured into a number of different forms and preparations; and, after all, they must be combined together, to form pills, powders, mixtures, &c., without end. Of course, all this adds to the mystery of the treatment of disease: and is not such mystery considerably enhanced, by clothing our prescriptions in an unknown tongue and unknown characters?

It has been remarked that "the whole tendency of empirical art is to busy itself in technicalities, and to place its pride in particular short cuts and mysteries known only to adepts; to surprise and astonish by results, but conceal processes." Has the profession itself then but been supplying lessons to the acknowledged quack? Alas, we fear we must confess ourselves herein guilty. We have not been prescribing medicines like philosophers, we have been only rudely and blindly experimenting with drugs. We have been engaged in what Bacon calls the "anticipation of nature," instead of the "interpretation of nature." We have forgotten his wise and weighty aphorism, which, however trite, cannot be too often sounded in our ears: "Man, the minister and interpreter of nature, can act and understand in as far as he has either in fact or in thought observed the order of Nature; more he can neither know nor do."

These remarks are intended to introduce a few observations which I have to make on the Natural History and Treatment of Wounds. In the case of an external injury, we are invariably met with the demand, what application will heal or cure it? In former days they had sarcotic or flesh-creating ointments, and in our own days we have *healing cerates*, and other similar preparations without number.

Let us first inquire what Nature can accomplish in the matter of wounds; and then, how she may be aided in her operations. Dr. Macartney states, that as to the effects of injury in the different classes of animals, he found, "that the powers of reparation and reproduction are in proportion to the indisposition or incapacity for inflammation, and hence that inflammation is so far from being necessary to the separation of parts, that in proportion as it exists the latter is impeded, retarded, or prevented; and that when inflammation does not exist, the reparative power is equivalent to the original tendency to produce and maintain organic form and structure; that it then becomes a natural function, like the growth of the individual or the reproduction of the species." This is quite different from the doctrines formerly taught under the terms *adhesive, suppurative, ulcerative inflammations.* There is no countenance here given to Sir Astley Cooper's statement—"No wound can be repaired without inflammation."

Dr. Macartney describes the modes of reparation as follows:—1. Immediate union without any intervening substance such as blood or lymph. 2. The union by the medium of coagulable lymph, or a clot of blood. 3. The modelling process, or reorganization without any medium of lymph or granulations, the cavity of the wound becoming obliterated by a natural process of growth. 4. The reparation by means of a new, vascular, and organized substance, called granulations. In the treatment of wounds therefore, the great object of the surgeon must be to prevent inflammation, and thereby secure reparation by any of the first three modes; if he is successful in this object, granulation and suppuration, which go together, will be obviated. The following simple rules seem to embrace all that is necessary to facilitate nature's operations:—approximate the edges of the wound gently and without much traction (after having cleaned it and removed foreign bodies); use as few stitches as possible; use as little adhesive strap as possible; apply a pledget of cloth soaked in cold water, and bandage loosely; inculcate absolute rest; preserve the part moist and cool, by the assiduous changing of cloths wrung out of cold water, and applied over the bandage; the part must not be allowed to become heated, so that for the first few days the cloths must be changed every two or three minutes, or a minute continuous stream must be directed on the part, by any of the simple processes recommended for the purpose. By the use of the cold water dressings, incised wounds heal immediately, and lacerated wounds detach sloughs, and are repaired by the modelling process without suppuration, at the same time presenting the most excellent cicatrix. In the latter kind of wounds, when poulticing is used, profuse suppuration is established, inflammation being excited by the hot, rancid, oppressive, irritating poultice, much of the previously sound tissues are wasted away, and the resulting cicatrix is rigid, puckered, and contracted.

We read that Hippocrates himself used water dressing most successfully, but that afterwards Celsus introduced a variety of absurd and complicated medicines. In the 14th century, the system of secret dressing was in fashion, each practitioner having a remedy which he considered universally applicable. When at a still later period water dressings were used, they were accompanied with incantations, to which the good effects were attributed. It is stated that Ambrose Paré, a pious but superstitious man, used the same application, but astonished at his extraordinary success, deemed the remedy nothing less than miraculous, and therefore not to be used by mortals, and accordingly he abandoned it.

This mode of treating wounds has received at my hands a very extensive trial, and has been followed with great success. I must, however, confess that I have had no inconsiderable difficulty in overcoming the prejudices of the people against so simple a method; and, in ordinary private practice, it is not unlikely I might have been obliged to discontinue it, or at any rate, substitute the usual more formal perfumed lotions; but the nature of my appointments in connexion with the extensive manufactories in this district, has enabled me to carry forward the simpler practice, and that too, at last, to the entire satisfaction of the people themselves. Such prejudices are not confined to our locality, and have been, I fear, too much fostered everywhere by the more mysterious proceedings of the scholastic and orthodox practitioners. If this is so, we need be less astonished at the success of quackery, which is conducted upon similar principles. When we let all our patients see and comprehend that we are treating them upon scientific and simple principles, then may empiricism prepare for its downfall without any interference on the part of government.

I shall conclude with a brief note of a few cases which have occurred recently, in illustration of the foregoing observations.

I. A man received an injury by the machinery in a large paper mill, which laid open the wrist-joint. The hand was half separated from the fore arm, the tendons were torn, and the inferior end of the radius, which is naturally related to the carpus, was exposed. The arm and hand were placed straight upon a pillow, the wound was cleaned, and two stitches taken; a pledget of cloth soaked in cold water was applied, and a bandage rolled, not too tightly, round the hand, wrist, and fore arm; a large basin of cold water was placed conveniently by the bedside, and directions left to apply freshly-soaked cloths over the bandage every two or three minutes, to prevent any heat or inflammation ensuing. No inflammation took place; the modelling process was uninterrupted, without suppuration, and an excellent cicatrix formed in little more than a fortnight.

II. A girl had the whole of the soft parts on the palm or surface of the four fingers, as it were, scraped off by the machinery in a flax mill; the tendons were torn, and the phalanges exposed at different places. Each finger was dressed as follows every day: being first bathed in cold water, a piece of soft cloth was placed round the finger, and a narrow roller to keep it applied; when the fingers were all thus dressed, a larger cloth soaked in cold water was wrapped round them together, and changed as frequently as the slightest tendency to become heated appeared. The modelling process advanced steadily without suppuration, and cicatrization was completed in about four weeks. The fingers gradually acquired flexibility.

A great number of similar accidents have occurred among boys and girls employed in the cotton and flax factories in this district during the last six or seven years; and the same simple treatment has been adopted, so that, although obliged occasionally to amputate fingers in part or in whole, cases of very remarkable injury of soft parts and bones have recovered, and members have been saved, which, in all likelihood, would have been sacrificed by a treatment less calculated to prevent inflammation and suppuration. Flabby granulations are seldom seen, unless where the prevention of inflammation is carelessly attended to; so that caustic applications, astringent lotions, and stimulant ointments are not used.

III. A little boy had scrofulous disease of the bones of the ankle-joint, on account of which I amputated, by the flap operation, below the knee. Two stitches were used for two days; a strip or two of plaster and cloths wrung out of cold water were the sole applications. The wound was whole in a week. Other amputations have been similarly treated, with equal success.

IV. A girl received a sharp instrument into the ball of the eye, at the Woodside works. The cornea and sclerotic coat were ruptured, the iris was lacerated, and prolapsus followed. Rest in bed, continued persevering use of cloths wrung out of cold water, and simple laxative medicine, constituted the treatment. The treatment was effectual in preventing inflammation, which was clearly the only indication in the case. The termination was as favorable as could be under such circumstances.

A multitude of cases might be recorded in this place in which the same simple natural treatment was adopted; but these instances suffice to show what Nature can accomplish herself, and the little we have to do to facilitate her operations.

